# MANAGEMENT OF INFANTILE BLOUNT'S DISEASE WITH MOLDED ORTHOSES: A NEW PERSPECTIVE

**DOI:** 10.1590/1413-785220162402153725

**Published:** 2016

**Authors:** Nei Botter Montenegro, Bruno Sergio Ferreira Massa, Luiz Renato Agrizzi de Angeli

**Affiliations:** 1. Universidade de São Paulo, Faculdade de Medicina, Hospital das Clínicas, Instituto de Ortopedia e Traumatologia, São Paulo, SP, Brazil

**Keywords:** Genu varum, Orthotic devices, Therapeutics, Braces

## Abstract

**Objective:**

: This retrospective study evaluated treatment with individually contoured molded bracing at early stages of the disease.

**Methods:**

: We evaluated the medical records of patients undergoing treatment of Blount's disease with molded orthoses for medial decompression between 2010 and 2014. The deformity angle (Drennan's metaphyseal-diaphyseal angle) and Langenskiöld classification were measured before and after treatment by a pediatric orthopedic surgeon with over 5 years of practice, blinded for the study and patients.

**Results:**

: The mean age was 2.57 years old. Four patients were female and six male. Half of the total sample had bilateral disease. The average deformity angle showed a statistically significant reduction after treatment (p <0.001). Gender and laterality did not statistically influence the change of the deformity angle after treatment (p> 0.05).

**Conclusion:**

: The nightly use of molded orthoses for medial decompression was effective in reducing the metaphyseal-diaphyseal angle in Blount's disease in children under 3 years of age, regardless of gender and bilateral disease. Patients over 3 years old did not benefit from bracing. Level of Evidence IV, Case Series.

## INTRODUCTION

Blount's disease, identified and described by Blount in 1937^1^ and initially reported by Erlacher^2^ in 1922 is a condition of unknown etiology, characterized by varus deformity^3^ of the proximal tibia in previously healthy children, usually between two and five years old.^2^^,^ 4

Due to the risk of evolving with progressive angular deformity, many cases come to require surgical treatment for correction and re-establishment of lower limb alignment.^5^^-^ 14 In the early stages of the disease, however, several authors have proposed treatment with orthotics.^7^^,^ 10 ^,^ 11 ^,^ 15 ^-^ 21 The indications include children under the age of three years and unilateral disease Langenskiöld stage I or II.^22^^,^ 23 Patients over four years old, with weight to age percentile > 90, presenting bilateral disease or progressive deformity did not show good performance with nonsurgical treatment.

Classically described in articles and textbooks, however, the use of orthoses had its effectiveness evaluated in a few papers, which limited its indication and generated controversy both regarding application and functionality. The difficulties encountered by researchers include: 1) lack of standardization of orthotic devices; 2) difficulty of regulating the treatment regimen; 3) absence of control groups; and finally, 4) the differentiation between Blount's disease and physiological *genu varum*.^19^^,^ 23

The latest study assessing conservative treatment with orthoses dates back over 10 years. The indication of conservative treatment is based on papers published in the late 90s,^19^^-^ 21 mainly guided by Richards' study,^20^ as explained by Birch in the latest review on the subject, published in the Journal of the American Academy of Orthopaedic Surgeons.^22^

In our service, we have been using a new orthosis that is clinically efficient. This retrospective study aimed to evaluate treatment with this type of individually molded orthosis in children presenting early stages of the disease. 

## METHODS AND CASES

The medical records of patients undergoing treatment for Blount's disease with molded orthotics for lateral decompression between 2010 and 2014 were evaluated retrospectively. The project was approved by the Ethics Committee for Analysis of Research Project (CAPPesq) of *Hospital das Clínicas da Faculdade de Medicina da Universidade de São Paulo*,São Paulo, SP, Brazil under N° 13,248.

Inclusion criteria: Patients with confirmed diagnosis of Blount's disease younger than five years old at the beginning of treatment and Langenskiöld stage I to IV. Exclusion criteria: A diagnosis other than Blount's disease, non-adherence to treatment, patient's age higher than five years old at the beginning of treatment and Langenskiöld stage V or VI.

**Figure 1.**
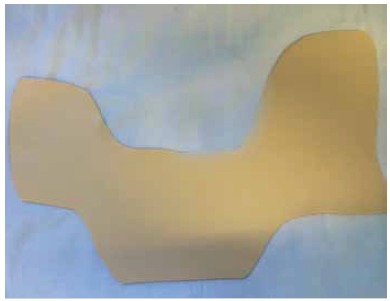
Orthoses mold.

**Figure 2.**
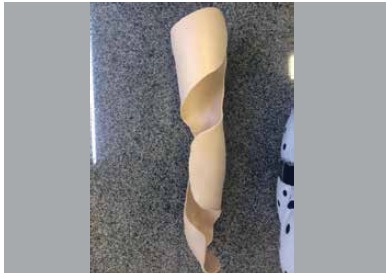
Example of orthoses molded for the left inferior limb.

All medial decompression orthotics were made by the occupational therapy team in low temperature thermoplastic material molded directly into the patient's leg and holding three full contact points. Thus, the orthosis applies forces at its proximal ends (positioned in the inner thigh),at the distal end of the tibia and at the medial and lateral fulcrum located at the proximal lateral end of the tibia. (Figures 1 and 2)

**Figure 3.**
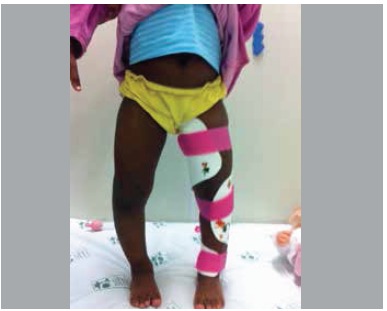
Child using molded orthoses left inferior limb. Observe the three full contact points (thigh inner edge, outer edge of the proximal tibia, and distal inner edge of the tibia), promoting decompression on the medial proximal edge of the tibia.

This design results in a non-articulated single device which incorporates all the forces. Modulated by the force applied to the fulcrum, the two momentum arms transmit the force to the proximal and distal support so that the orthosis exerts mechanical work similar to a bending test, however,with forces producing no rotational motion, but, due to their lateral positioning,exert decompression force on the inside edge of the knee. (Figure 3)

Patients and their responsible were instructed to use the orthosis only at night, during the patient's sleep and daily for at least 8h per day. We did not recommend its use in morning or afternoon sleeping periods. From the start of treatment, we scheduled return appointments in one week, three weeks,six weeks and three months, in which the patient was assessed by the medical staff and the occupational the rapystaff.The medical evaluation was aimed at improving adherence to treatment, as well as to follow the deformity clinically and radiologically.

The occupational therapy team was in charge of making adjustments and eventual repairs to orthoses. After the 3^rd^ month post-operatory consultation, returns were scheduled every three months. Panoramic radiographs of the lower limbs were indicated at each consultation from the 3^rd^ month. The proposed follow-up time was one year and may have been extended to two years if there was progressive improvement of the Drennan's metaphyseal-diaphyseal angle. Each orthoses had an average cost of R$ 140 (around US$ 35) and needed to be replaced every six months, due to the child's growth.

### Radiographic evaluation

The deformity angle (Drennan's metaphyseal-diaphyseal angle)^21^ and Langenskiöld classification in panoramic anteroposterior radiographs of the lower limbs before and after treatment were blindly assessed by an orthopedic surgeon specialized in pediatric musculoskeletal disorders, with over five years of practice.

### Statistical evaluations

The angles were evaluated before and after treatment with summary measurements as with generalized estimating equations with exchangeable matrix correlations between times, and with marginal Normal distribution and identity link function.^24^

Additional assessments were conducted between unilateral and bilateral groups, gender (male and female) and age (older and younger than three years old) using estimating equations generalized by interchangeably matrix correlations between sides, with marginal Normal distribution and identity link function.

Ages have been reported with summary measures (mean, standard deviation, median, minimum and maximum) and the other qualitative characteristics of the patients were described as absolute and relative frequencies.^25^

Any reduction in the Langenskiöld scale and in the classification after treatment were considered as an improvement; then,the association of the patients' characteristics with scale improvement was verified with generalized estimating equations with exchangeable matrix correlations between moments with binomial distribution and logit link function.^24^

The tests were performed at 5% significance level.

## RESULTS

Medical records from sixteen patients were initially separated for analysis, totaling 27 tibiae. Of these, six patients (12 tibiae) did not meet the inclusion criteria, all due to lack of X-rays in the digital imaging system of our service or in the medical records. Thus, there were 10 patients (15 tibiae) for final analysis.

The mean age was 2.57 years old (eight patients were less than three years old and two over three years old). Regarding gender, four patients were female and six male. Half of the total sample had bilateral disease. (Table 1)

**Table 1.** Description of the patient's characteristics.

**Variable**	**Description (N = 10)**
Age (years old)	
Mean (St. Dev.)	2.57 (0.99)
Median (min / max)	2.21 (2; 5)
Age range, n (%)	
< 3 years old	8 (80.0)
≥ 3 years old	2 (20.0)
Gender, n (%)	
Feminine	4 (40.0)
Masculine	6 (60.0)
Laterality, n (%)	
Unilateral	5 (50.0)
Bilateral	5 (50.0)

The patients deformity angle showed statistically significant average reduction after treatment (p<0.001). (Table 2)

**Table 2.** Description of deformity angles and Langenskiöld scale classification according to the moment of assessment and result of comparison between moments.

**Variável**	**Moment**	**p**	
	**Initial (N = 15)**	**Final (N = 15)**	
Angle			<0.001
Mean (St. Dev.)	14 (6.2)	7.7 (5.6)	
Median (min/max)	14 (6; 32)	8 (0; 19)	
Langenskiöld scale, n (%)			0.179
1	3 (20.0)	10 (66.7)	
2	9 (60.0)	2 (13.3)	
3	2 (13.3)	3 (20.0)	
4	1 (6.7)	0 (0.0)	

In the comparison between patients younger and older than three years, the correction was more effective in children under three years old (p = 0.009). Patients aged three years or older showed, on average, angle increase after treatment. Gender and laterality did not statistically influence the change of the deformity angle after treatment (p> 0.05). (Table 3)

**Table 3.** Description of changes in deformity angles after treatment according to the patient's characteristics and results of the comparisons.

**Variable**	**Mean(St. Dev.)**	**Median (min/max)**	**N**	**p**
Age range, n (%)				0.009
< 3 years old	8.2 (6.1)	8 (0; 24)	12	
≥ 3 years old	-1.3 (2.1)	-2 (-3; 1)	3	
Gender, n (%)				0.563
Feminine	7.6 (9.5)	6 (0; 24)	5	
Masculine	5.6 (5.3)	8 (-3; 12)	10	
Laterality, n (%)				0.902
Unilateral	6.6 (10.9)	2 (-3; 24)	5	
Bilateral	6.1 (4.2)	7 (-2; 12)	10	
Total	6.3 (6.7)	6 (-3; 24)	15	

There was improvement in Langenskiöld classification scale after treatment, which, however, was not statistically significant (p = 0.179). (Table 4)

**Table 4.** Description of improvement in Langenskiöld classification according to the patient's characteristics and result of associations.

**Improvement in Langenskiöld classification**				
**Variable**	**No n (%)**	**Yes n (%)**	**Total n (%)**	**p**
Age range, n (%)				0.113
< 3 years old	5 (41.7)	7 (58.3)	12	
≥ 3 years old	2 (66.7)	1 (33.3)	3	
Gender, n (%)				0.715
Feminine	2 (40.0)	3 (60.0)	5	
Masculine	5 (50.0)	5 (50.0)	10	
Laterality, n (%)				0.379
Unilateral	3 (60.0)	2 (40.0)	5	
Bilateral	4 (40.0)	6 (60.0)	10	
Total	7 (46.7)	8 (53.3)	15	

## DISCUSSION

Blount's disease is diagnosed in most cases between one year and a half to three years old, mainly associated with overweight and children with early history of deambulation.^20^ The differentiation between physiological *genu varum* and the disease itself is still matter of discussion.

There are few practical criteria for distinguishing the two entities. The physiological *genu varum* resolves itself usually up to two years old and presents a Drennan's metaphyseal-diaphyseal angle<9°.^21^ To exclude these cases from the study, only cases of patients older than two years at the beginning of treatment were included. Moreover, the average Drennan's meta-diaphyseal angle was 14°, ranging between 6° and 32° at the start of treatment. The inclusion of patients also took into account the clinical criteria of non-improvement or worsening of the deformity after two years old, which lead us to include two patients with baseline measures deformity angle <9° in the study.

**Figure 4.**
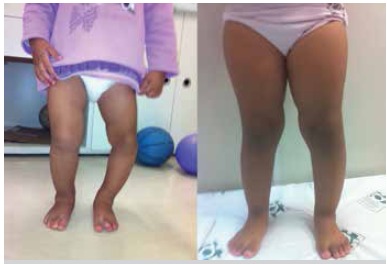
Clinical photos before (left) and after (right) treatment with orthosis in a patient with Blount's disease to the left.Note the correction of the genu varum six months after treatment.

**Figure 5.**
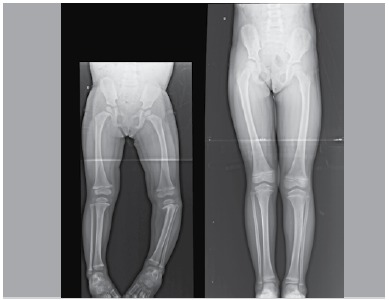
Panoramic x-ray before (left) and after (right) treatment with orthosis of a patient with Blount's disease to the left.

The use of orthoses was effective in reducing Drennan's metaphyseal-diaphyseal angle (initial mean x,final mean y), with statistical significance. (Figures 4 and 5) Only three tibiae (20% of the total sample) showed no reduction of the deformity angle and two of them (13%) were from patients older than three years. Even without full correction (a varus deformity was maintained in most cases) only cases with worsened deformity underwentsurgery on follow up. Even if it was necessary, an eventual surgery would benefit of some angular correction. This, in itself, would be a good indication for orthosis. Another important factor would be no progression of the deformity in patients with appropriate indication.

It was observed that patients younger than three years evolve better than patients older than three years, which is in agreement with treatment indications published in the latest reviews on this topic.^22^^,^ 23 Moreover, our study found no difference in evolution related to gender, making both boys and girls eligible for the treatment.

Our study was limited to a small sample size and its retrospective design. The first is justified by the low incidence in the general population and limitations of the public health system that redirects patients at more advanced stages of the disease and older patients. Despite this scenario, the improvement of the meta-diaphyseal angle was significant, favoring the use of orthosis.Regarding the retrospective nature of the study, we are planning new prospective and randomized steps.

Current treatment indications with orthoses encompass children under three years old with unilateral disease Langenskiöld stage I or II.^22^^,^ 23 This is based on the best results obtained so far, demonstrated by Richards et al.^20^ in 1998. In this study, the children used orthosis during the day, and were not encouraged to use them during the night, because then, it was believed that orthosis should be used while walking, i.e., when the limbs were subject to stress in varus. However, the bilateral daytime use substantially hinders deambulation, reducing adherence to treatment and, perhaps for this reason, bilateral cases did not evolve as well as unilateral cases.

Raney et al.^21^ found no difference between the clinical improvements of patients who used the orthosis only at nighttime and those who used it continuously, suggesting that nighttime use would be sufficient to correct the deformity and also facilitate adherence to treatment, allowing good progress of cases with bilateral disease. We believe that this approach is superior to the continuous or daytime use, since we obtained favorable results in the correction of deformity with nighttime use. We found no difference between unilateral and bilateral cases (both showing significant improvement) in addition to saving the child from adverse psychological and social effects of daytime use, which can harm their development.^21^ This approach facilitated adherence to treatment, after all, patients were not excluded from our sample for lack of adhesion, although we do not have objective data to confirm it.

Another parameter observed in our study was radiographic improvement of Langenskiöld classification. This classification is helpful in the initial evaluation of the patient, but the main studies and reviews do not mention that the use of orthoses improves Langenskiöld classification, even though deformity angle improved.^19^^-^ 23 We did not find statistically significant differences in the evaluation of Langenskiöld classification before and after treatment, despite the significant reduction in Drennan's metaphyseal-diaphyseal angle.

We recommend reproducing the treatment with medial decompression molded orthoses for the treatment of Blount's disease in patients younger than three years presenting Langenskiöld classification I and II.

## CONCLUSION

The nighttime use of medial decompression molded orthoses was effective in reducing the meta-diaphyseal angle in Blount's disease in children younger than three years of age, regardless of gender and disease laterality. Patients over three years old did not benefit from orthoses. 

There was improvement in Langenskiöld classification especially in groups 1 and 2. This improvement, however, did not reflected statistical significance (p = 0.179).
