# Erratum

**DOI:** 10.1590/1413-785220162402153725ER

**Published:** 2016

In the article MANAGEMENT OF INFANTILE BLOUNT’S DISEASE WITH MOLDED ORTHOSES: A NEW PERSPECTIVE, DOI: http://dx.doi.org/10.1590/1413-785220162402153725, published in the journal *Acta Ortopédica Brasileira*, 24(2):85-9, 2016, on page 85 where it reads:

“Nei Botter Montenegro^1^, Bruno Sergio Ferreira Massa^1^ , Luiz Renato Agrizzi de Angeli ^1^” 

it shall read:

“Maria Cândida de Miranda Luzo^1^, Nei Botter Montenegro^1^, Bruno Sérgio Ferreira Massa^1^, Luiz Renato Agrizzi De Angeli ^1^, Felippi Guizardi Cordeiro^1^, Roberto Guarniero^1^” 

on page 88 where it reads:

“ACKNOWLEDGEME The authors wish to thank Felippi Guizardi Cordeiro, Maria Cândida de Miranda Luzo, Patricia Moreno Grangeiro, Roberto Guarniero and Rui Maciel de Godoy Junior for their intellectual contribution to the development of the concept of this study.”

it shall read:

“ACKNOWLEDGEME The authors thank Patrícia Moreno Grangeiro and Rui Maciel de Godoy Junior for their intellectual contribution on the development of the study.”

on page 89 where it reads:

“AUTHORS’ CONTRIBUTIONS: Each author contributed individually and significantly to the development of this study. BSFM (0000-0002-3335-8661)* and LRAA (0000-0003-3779-6914)* were the main contributors on writing the manuscript. BSFM followed the patients and collected clinical data. NBM (0000-0002-0705-1623)* performed the measurements and classifications inherent to the study.*ORCID (Open Research and Contributor ID).”

it shall read:

“AUTHORS’ CONTRIBUTIONS: Each author contributed individually and significantly for the development of the study. BSFM (0000-0002- 3335- 8661)* and LRAA (0000-0003-3779-6914)* wrote the manuscript. BSFM and FGC (0000-0002-0175-2912)* followed up the patients and collected clinical data. The Occupational Therapist MCML (0000-0003-3565-770x)* designed and made the orthoses used in the study. NBM (0000-0002-0705- 1623)* performed the measurements and classifications inherent to the research. RG (0000-0002-1798-0653)* reviewed the manuscript and contributed to the intellectual concept of the study. *ORCID (Open Research and Contributor ID).”

All content of the journal, except where identified, is licensed under a Creative Commons attribution type BY-NC.

